# Temporal-Spatial Correlation between Angiogenesis and Corticogenesis in the Developing Chick Optic Tectum

**DOI:** 10.1371/journal.pone.0116343

**Published:** 2015-01-29

**Authors:** Alejandra Rodriguez Celin, Melina Rapacioli, Mariela Azul Gonzalez, Virginia Laura Ballarin, Sara Fiszer de Plazas, Juan José López-Costa, Vladimir Flores

**Affiliations:** 1 Interdisciplinary Group in Theoretical Biology, Dep. Biostructural Sciences, Favaloro University, Buenos Aires, Argentina; 2 Institute of Cell Biology and Neurosciences “Prof. E. De Robertis”; Buenos Aires University-CONICET, Buenos Aires, Argentina; 3 Digital Image Processing Group, School of Engineering, National University of Mar del Plata, Mar del Plata, Argentina; Medical College of Wisconsin, UNITED STATES

## Abstract

The developing chick optic tectum is a widely used model of corticogenesis and angiogenesis. Cell behaviors involved in corticogenesis and angiogenesis share several regulatory mechanisms. In this way the 3D organizations of both systems adapt to each other. The consensus about the temporally and spatially organized progression of the optic tectum corticogenesis contrasts with the discrepancies about the spatial organization of its vascular bed as a function of the time. In order to find out spatial and temporal correlations between corticogenesis and angiogenesis, several methodological approaches were applied to analyze the dynamic of angiogenesis in the developing chick optic tectum. The present paper shows that a typical sequence of developmental events characterizes the optic tectum angiogenesis. The first phase, formation of the primitive vascular bed, takes place during the early stages of the tectal corticogenesis along which the large efferent neurons appear and begin their early differentiation. The second phase, remodeling and elaboration of the definitive vascular bed, occurs during the increase in complexity associated to the elaboration of the local circuit networks. The present results show that, apart from the well-known influence of the dorsal-ventral and radial axes as reference systems for the spatial organization of optic tectum angiogenesis, the cephalic-caudal axis also exerts a significant asymmetric influence. The term cortico-angiogenesis to describe the entire process is justified by the fact that tight correlations are found between specific corticogenic and angiogenic events and they take place simultaneously at the same position along the cephalic-caudal and radial axes.

## INTRODUCTION

The developing chick optic tectum (OT) is a widely used model of both corticogenesis and angiogenesis [1–11]. Consistent descriptions of OT corticogenesis can be found in the literature [3–11]. By contrast, discrepancies are frequent in the literature about the OT angionenesis. As examples, different vascular bed organization and vessels displaying different degrees of differentiation have been described at the same embryonic day (ED) or developmental stage (DS) [1, 2, 12]. Besides, while some authors describe that vessel formation and differentiation progress from the pial to the ventricular surface [2, 13–15], others describe that, in the forebrain, vessels growth progresses in the opposite direction [16].

These discrepancies have prevented a coherent morpho-histogenteic interpretation and a unified comprehension of corticogenesis and angiogenesis in the developing OT. By contrast, in others species, simultaneous analyses of both processes have demonstrated that the stratification of the cerebral cortex is accompanied by the emergence of layer-specific characteristics in the vascular beds [17–18].

The variability found in OT angiogenesis versus the consistency observed in OT corticogenesis is difficult to understand considering that: (**a**) the developing neural tissues adapt the developing vascular bed to its own 3D spatial organization [19–22] and that (**b**) angiogenesis and neurogenesis are interactively regulated by sharing several regulatory mechanisms [23, 24].

It is likely that the above mentioned discrepancies on the OT angiogenesis result from the fact that, sometimes, the asymmetrical development of the OT is not taken into account. It is known that the OT corticogenesis progresses according to a developmental gradient axis extended from the cephalic-lateral-ventral region to the caudal-medial-dorsal region [3, 5, 25]. Coherently, the OT angiogenesis also progresses asymmetrically according to: (**a**) a ventral-to-dorsal axis [26] and (**b**) a radial (ependimal-to-pial) axis. By contrast, there is a complete absence of studies reporting a cph-cd asymmetry in OT angiogenesis.

A dynamic table of OT corticogenesis, as a function of time and space, was recently introduced to explain how OT regions located at different positions along the cephalic-caudal (cph-cd) axis consistently display different DSs [10]. This table shows that DSs progresses as a function of the time and also propagate along the cph-cd axis. This is the reason why several different DSs are found along the cph-cd axis at the same ED or the same embryonic Hamburger and Hamilton [27] stage (HH stage).

Given that the OT corticogenesis is spatially organized along the cph-cd axis [10], it is reasonable proposing that this asymmetry should have a polarizing influence on angiogenesis. If this proposal were correct, significant differences in vessels differentiation and spatial organization should be found along the cph-cd axis. In other words, at any ED or any HH stage, the developing vascular bed should display a cph-cd polarized organization (a delayed DS at the caudal end and an advanced DS at the cephalic end).

The OT lamination progresses from a single layered neuroepithelium to a multilaminated cortical organization. The entire process implies progressing through laminated patterns of increasing complexity and the present paper analyzes the effects of such changing patterns on the developing vascular network. In order to find out spatial and temporal correlations between corticogenesis and angiogenesis, this work tends to ascertain whether the increase in complexity observed during the OT angiogenesis can be appropriately described within the frame provided by the table of DSs that describe the OT corticogenesis. With this purpose, several methodological approaches were applied to analyze the dynamic of angiogenesis in the developing chick OT from an early stage when the OT is an avascular neuroepithelium up to the time when the OT cortex acquires its basic cortical histo-architecture.

## MATERIALS AND METHODS

### Animals

Pathogen-free fertilized *White leghorn* chicken eggs were obtained from Rosenbusch Institute (Buenos Aires). They were incubated at 39°C and 60% relative humidity. Animals were treated according to the Guide for the Care and Use of Laboratory Animals (Institute of Laboratory Animals Resources, Commission of Life Sciences, National Research Council) and procedures were approved by the Laboratory Animal Care and Use Committee of the Favaloro University. Ten Embryos were studied every day between the 2^nd^ and the 12^th^ EDs.They were anesthetized by hypothermia, removed from the eggs and staged according to the HH stages. Complete embryos (ED2-ED5/HH13-HH28) or brains (ED6-ED12/HH29-HH38) were dissected out in ice-cold 0.1 mol l^-1^ sodium phosphate buffer (pH 7.4), plus 0.9% w/v NaCl (PBS) and then fixed by immersion in 4% paraformaldehyde in PBS at room temperature (RT). The time of fixation varied from 40 minutes (ED2 embryos) to 4 hrs (ED12 brains).

Three groups of embryos were used to obtain: (a) whole mount preparations (n = 33 embryos), (b) complete histological serial sections for conventional staining (n = 22 embryos), and (c) serial sections for immunocytochemical staining or diaphorase technique (n = 55 embryos).

### Histology

For conventional histology, after fixation, specimens were washed in PBS, dehydrated in graded ethanols, cleared in xylene and embedded in Paraplast at 56°C (Product No. 8889–501006. Pellet form). Four OT were studied on each ED. Serial 10 µm thick sections performed according to different spatial orientations were obtained (See Morphometry). They were rehydrated in a decreasing ethanol gradient and stained with hematoxylin and eosin. Then, they were dehydrated and mounted with Histomount (National Diagnostics, Inc. HS-103) on standard slides. Whole mount embryos and complete serial sections of embryos of defined EDs and HH stages purchased from Carolina Biological Supply Company were also analyzed in this study.

### Diaphorase

For the analysis of vascular development, the vascular tree was revealed by the diaphorase technique. After fixation, midbrains were washed with PBS and embedded in 4% agarose (A9414, Sigma) in PBS. Six OT were used on each ED. Serial 100-µm thick sections were obtained with an Oxford vibratome. Sections were incubated for 1 hour at 37°C in a solution containing 0.1% NADPH and 0.02% nitrobluetetrazolium diluted in 0.1 M PBS with 0.3% Triton X-100. Then they were mounted with polyvinyl alcohol mounting medium with DABCO, antifading (10981, Fluka). This technique also labels neurons expressing nitric oxide synthase [28]. Six adjacent sections from each OT were used to obtain quantitative data (number and position of vessels branches and bifurcations).

### Immunocytochemistry

Sections coinciding with the cph-cd axis (See Morphometry) were immunolabeled with the primary antibodies listed in Table 1. Following fixation, specimens from ED2 to ED12, were dehydrated and embedded in paraffin. Ten µm thick serial sections (four OT on each ED) were obtained and collected on gelatinized slides, dried for 1 hour at 37°C and then stored at 4°C. Before immunostaining, sections were deparaffinized, rehydrated, rinsed in PBS and processed for antigen retrieval. Nonspecific binding was blocked by preincubating the sections in 5% normal goat serum (NGS) in PBS with 0.05% Triton X-100 (TX-100) for 1 hour at RT in humidity chamber.

**Table 1:** Primary antibodies characteristics. .

**Antibody/species**	**Supplier/code/lot**	**Immunogen**	**Dilution**
Rabbit polyclonal antinotch homolog 1, translocation-associated (Notch1)	Lifespan biosciences LS-C16928 Lot 6113061	Synthetic peptide CQHSYSSPVDNTPSHQ N-terminal added cysteine Human notch1 intracell. domain (aa 2488–2502)	1:300
Rabbit polyclonal antineurod (NeuroD1)	Lifespan biosciences LS-C9245 Lot 8030526	Synthetic peptide DDDQKPKRRGPKKKKM Conjugated to malemide-activated KLH	1:100
		Human NeuroD1 (aa 76–91)	
Mouse monoclonal antineuron specific ßeta III tubulin [TUJ-1]	Abcam ab14545 Lot 543886	Rat brain microtubules (whole Protein)	1:500

These sections were used for immunolabeling with the antibodies directed to Notch1, NeuroD1 and ß-III-Tubuline (ßIIITub). Immunolabeling was performed with primary antibodies diluted in PBS containing 0.5% NGS (See Table 1). Sections were incubated with the primary antibodies for 20 hours at 4°C in humidity chamber. After several rinses in PBS, sections were incubated with secondary antibodies diluted 1:1,000 in PBS for 2 hours at RT in a dark humidity chamber. Sections were then rinsed in PBS and counterstained with nuclear dye Hoechst 33342 (B-2261, Sigma) in PBS (dilution 1:1,000) at RT in a dark humidity chamber for histoarchitecture analysis. After rinsing, slides were mounted with polyvinyl alcohol mounting medium with DABCO, antifading (10981, Fluka). Alexa Fluor 488 goat anti-rabbit IgG (H1L) (A-11008, Molecular Probes) and Alexa Fluor 488 F(ab’)2 fragment of goat antimouseIgG (H1L) (A-11017, Molecular Probes) were used as secondary antibodies.

**Antigen retrieval.** Antigen retrieval for Notch1 was performed by treatment with 0.1% TX-100 in PBS for 30 minutes at RT with gentle rotary shaking. ßIIITub retrieval was performed by treatment with 0.294% sodium citrate, pH 6 for 15 minutes at 95°C. NeuroD retrieval was performed by treatment with 0.1% citric acid, pH 2.7 for 15 minutes at 95°C.

**Controls.** The antibodies used in this paper were used as biomarkers for different cell elements: arterial- type endothelial cells, neuroepithelial (NE) cells bodies, nuclei and processes as well as neuronal perikarya and/or nuclei, developing neurites (dendrites and axons). Negative controls for the primary antibodies were performed by using several chick adult non-neural and nonendocrine tissues. Negative controls for the secondary antibodies were performed on OT sections of each ED processed without preincubation with primary antibodies. Neither kind of negative control exhibited detectable fluorescence.

### Morphometry

Histological sections with different orientations were used depending on the ED, since the OT developmental gradient axis orientation changes during development [3, 5, 29, 30]. During the early stages (up to ED6) the OT anatomical longitudinal axis almost exactly coincides with cph-cd axis and slightly deviates from the developmental gradient axis orientation. Afterwards, the OT ‘rotates’ with respect to the neural tube axis and the anatomical longitudinal axis extends from the site where the retinal axons enter the OT to the caudal end. Sections with these orientations closely approximate the developmental gradient axis. Planes of section showing the maximal difference in tissue differentiation between both OT ends were considered as those that best approximate the developmental gradient axis position. For simplicity, planes of section coinciding with the developmental gradient axis are named as cph-cd sections. Four OT were studied on each ED.

### Data recording

Data was recorded and analyzed with an image processing device (Carl Zeiss, Oberkochen, Germany) consisting of an Axioplan 2 imaging optical epifluorescence microscope with an Axiocam HR color digital scanner and a computer equipped with the following software: Axiovision (Carl Zeiss, Oberkochen, Germany), MATLAB (The MathWorks, Inc., Natick, Massachusetts), 3ds Max (Autodesk, Inc. San Rafael, CA), CorelDRAW (Corel Corporation, Ottawa), Photoshop (Adobe Systems, San Jose, CA) and software implemented by our own group (IGTB, UF) [31, 32]. Brightness and contrast levels of digital images were adjusted by using the same set of specifications for images obtained at the same magnification.

OT DSs recording and constructions of 2D maps of DSs distribution along the OT cph-cd axis were performed. First, the OT DSs found along the entire cph-cd axis were recorded. According to the progress of corticogenesis the DSs were identified and demarcated in the histological sections, based on criteria given in [10].

Qualitative features (structural changes observed by means of conventional and diaphorase staining and immunolabeling) and quantitative parameters (number and position of vessels branches and bifurcations) were taken into account to globally describe the progression of angiogenesis in the developing OT. Six adjacent sections from each OT were used for quantitative analyses.

**Figure 1:**
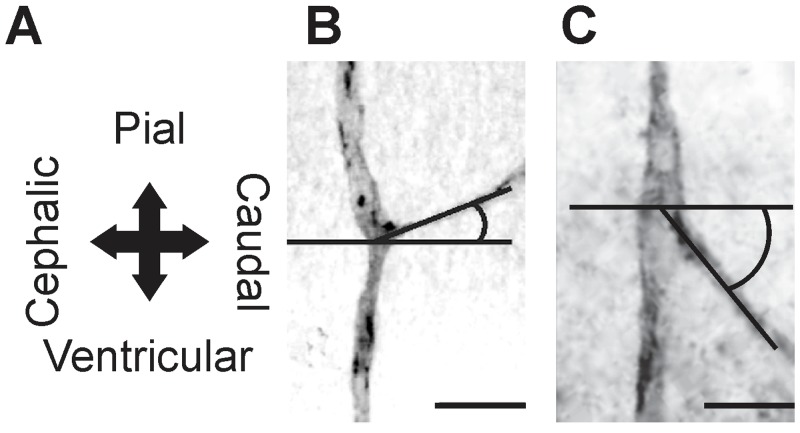
Recording of the angles that define the vessels growth direction. (A) Spatial reference axes of the OT cortex. (B) Ascending (pial-caudal) collateral branches. (C) Descending (ventricular-cephalic) collateral branches (Bars: 20 µm).

**Figure 2:**
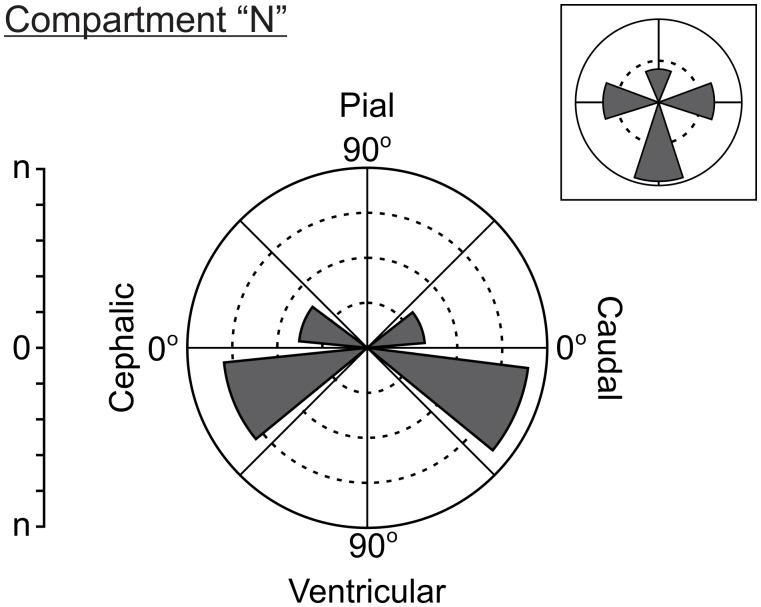
Polar coordinates system used to visually illustrate and quantitatively represent the directions of growth of collateral branches. The horizontal line (0°) coincides with the tangential plane of the OT cortex while the vertical line (90º) coincides with the radial (pial-ventricular) axis. The polar coordinates system is divided into 8 (45º amplitude) intervals. For each interval, a bar indicates the absolute number of collateral branches whose direction of growth is included within the interval. In order to simplify the information, inbox represents the number of collateral branches corresponding to 4 (90° amplitude) intervals.

A computer-assisted morphometric analysis was used to estimate the following parameters:

(a) The density of radial vascular sprouts that invade the OT neuroepithelium.
(b) Relative frequency (RF) of lateral and terminal branches of radial vessels as a function of both the position along the radial axis and the DS. The number of branches was counted in successive 10 µm length windows along the radial axis taking as reference (0 µm) the ventricular surface. These data are presented as bar graphs where bars represent the RF of lateral or terminal branches found in spatial windows located at different positions along the radial axis.
(c) Direction of growth of lateral and terminal branches of radial vessels. This parameter quantifies the preferential orientation of the developing vessels in terms of RF of vessels within a polar co-ordinates system where horizontal (0°) and vertical (90^o^) lines correspond to tangential and radial axes of the OT cortex respectively (Figs. 1 and 2). The polar co-ordinates system is divided into eight 45° amplitude intervals. The bar corresponding to each interval represents the RF of vessel whose spatial orientation belongs to that range of amplitudes. In order to simplify this information the ratio between vessels oriented in opposite directions (pial direction / ventricular direction; cephalic direction / caudal direction) was used as an index of isotropy. Values of this ratio significantly different from 1 indicate anisotropic distributions.

Algorithms for digital image processing based on mathematical morphology, skeletonization and Watershead transforms [33, 34] were used in order to analyze the following parameters:

(a) Estimation of the mean area of the tangential plane—measure on the pial surface- surrounding each radial vessel; this parameter is named as “perivascular area” and represents a kind of “area of influence” of each radial vessel.
(b) Characterization of the “shape” and the spatial pattern of distribution of perivascular areas over the OT tangential plane.

### Statistical analyses

**Number of embryos and sections.** One hundred and ten embryos (ten for each ED) were used. Within each ED, three embryos (six OT) were used for whole mount preparations, two embryos (four OT) for complete histological serial sections for conventional staining and five embryos for immunocytochemical staining (four OT) or diaphorase technique (six OT). Six adjacent 100-µm thick sections from each OT were used to obtain quantitative data. Given that OTs of each ED possesses more than one DS, the number of sections used for evaluate each DS varies between 72 and 180 (Table 2).

**Table 2:** Number of sections used to evaluate each developmental stage.

**Developmental stage**	**Embryonic day**	**Nº of ED**	**Nº of OT**	**Nº of sections/OT**	**Total Nº of sections/DS**
DS1	ED2, ED3, ED4, ED5, ED6	5	6	6	180
DS2	ED5, ED6, ED7, ED8	4	6	6	144
DS3	ED6, ED7, ED8	3	6	6	108
TS3–4	ED6, ED7, ED8, ED9, ED10	5	6	6	180
DS4	ED6, ED7, ED8, ED9, ED10	5	6	6	180
TS4–5	ED7, ED8, ED9, ED10	4	6	6	144
DS5	ED8, ED9, ED10, ED11, ED12	5	6	6	180
TS5–6	ED9, ED10, ED11, ED12	4	6	6	144
DS6	ED10, ED11, ED12	3	6	6	108
TS6–7	ED10, ED11, ED12	3	6	6	108
DS7	ED11, ED12	2	6	6	72

**Statistical tests.** Statistical analyses between different sets of data [(a) density of radial vascular sprouts that invade the OT, (b) relative frequency of lateral and terminal branches of radial vessels as a function of the position along the radial axis, (c) direction of growth of lateral and terminal branches of radial vessels or (e) perivascular and periarterial areas] corresponding to different DSs were implemented by means of the Statistics Toolbox (The MathWorks, Inc. MATLAB, Natick, MA). Statistical significance of the differences between two groups was determined by the Student’s “t” test. Statistical significance of the differences between multiple groups was determined by the one-way analysis of variance (ANOVA) followed by multiple comparisons of means using Tukey-Kramer post-hoc test. Correlations between the inter-vessel interval length and the position along the cph-cd axis were determined using the Pearson correlation coefficient. Linear correlations were assessed between inter-vessel interval length and the position along the cph-cd axis. Two-sample Kolmogorov–Smirnov test was used in order to compare probability density functions. Proportions were compared by means of Chi-square test and Fisher exact test. A value of p<0.05 was considered as statistically significant.

## RESULTS

During OT corticogenesis, the space between the inner limiting membrane (LM) (pial surface) and the outer LM (ventricular surface) of the neuroepithelium is gradually populated by different cohorts of neurons. Successive populations of migrating neurons are radially organized into transient neuronal layers or *transient cell compartments* (TCCs) [5]. The changing pattern of TCCs organization is determined by the way the migrating neurons are clustered during their radial migration. Each typical pattern of TCC organization defines a particular DS. Corticogenesis proceeds through successive DSs whose lamination pattern increases in complexity as a function of the ED. This increase in complexity derived from the fact that, as a rule, every newly defined TCCs derives by a process of segregation (cell sorting) from a preexisting one [5, 10].

### Qualitative description of the developing vascular network

**Figure 3:**
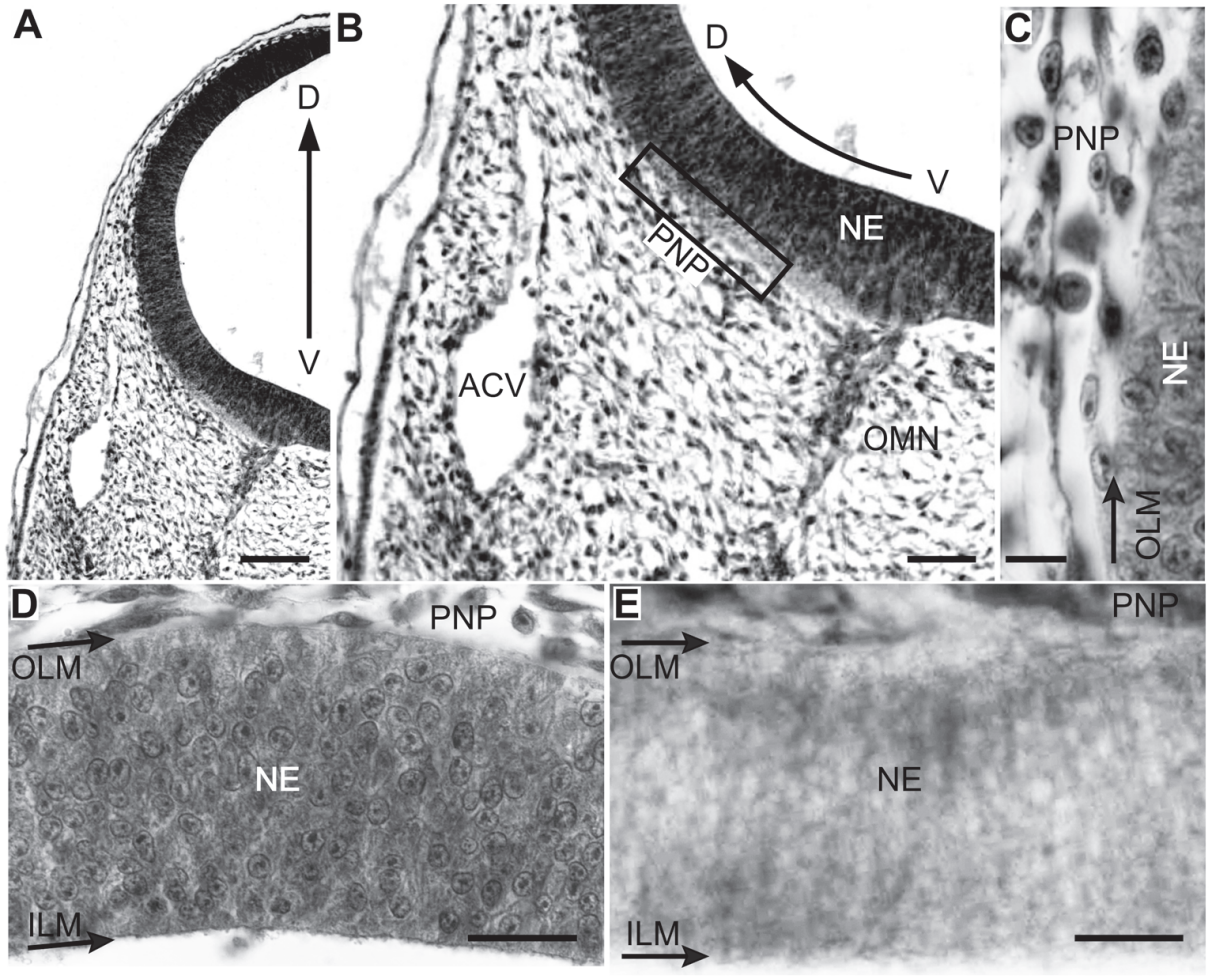
Radial organization of the OT vasculature during DS1 (ED3; HH20). (D-V sections; a-d: H-E; e: Diaphorase). (A-B) The OT is surrounded by the perineural vascular plexus (PNP). (C) Higher magnification of the box shown in b. Some endothelial cells contact the outer limiting membrane. (D-E) Endothelial cells are closely attached to the neuroepithelium basal surface but the OT wall is still deprived of vessels (Bars: A: 100 µm; B: 50 µm; C: 10 µm; D-E: 25 µm).

**Developmental Stage 1 (DS1)**. By ED2 an extrinsic leptomeningeal plexus, i.e., the perineural vascular plexus (PNP), appears over the ventral surface of the midbrain and rapidly, between ED2-ED3, expands toward the dorsal midbrain (Fig. 3, A-C). The DS1 is just beginning and the OT wall is an avascular single layered neuroepithelium composed of cylindrical neuroepithelial (NE) cells extended radially from the inner LM to the outer LM (Fig. 3, D and E).

**Figure 4:**
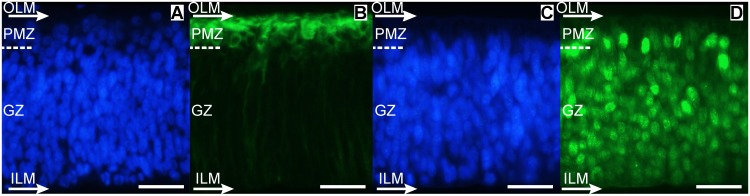
Radial organization of the OT cortex during DS1 (ED3; HH20). (A and C: Hoechst; B: β-III-Tubulin; D: NeuroD). The first post-mitotic neurons accumulate below the outer limiting membrane (OLM) and form an incipient premigratory zone (PMZ). They display the β-III-Tubulin+ cytoplasm labeling typical of the early differentiation neurons (B); they also display the intense NeuroD+ nuclear labeling that characterize the neuronal lineage (D). Dashed line: boundary between the generation zone (GZ) and the PMZ. (Bars: 20 µm).

**Figure 5:**
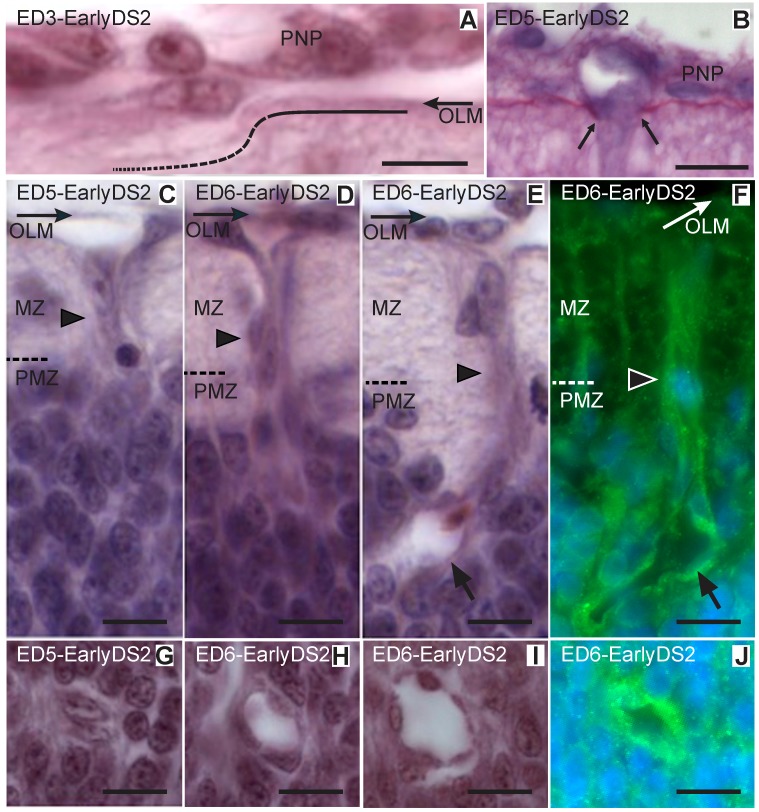
Ingression of primitive vessels into the OT (Early DS2; ED3–6; HH20–29). (A-D and F-H: H-E; E and I: Notch and Hoechst). (A) Endothelial cells attach to the outer limiting membrane (OLM) and degrade the basal membrane (dotted line). (B) PAS-Stained section reveals the interruption (between arrows) of the basement membrane at a site where a vessel sprouts traverses the pial surface and penetrates the neuroepithelium. (C) Solid sprouts (arrowhead) from the perineural vascular plexus (PNP) penetrate the neuroepithelium and pass through the marginal zone (MZ) toward the premigratory zone (PMZ). (D) Early primitive solid radial vessel (arrowhead) entering the PMZ. (E-F) After entering the PMZ the tip of the growing vessel develops a lumen (arrows). (G-J) Transverse sections of primitive radial vessels in different stages of development (Bars: A: 5 µm; B-J: 10 µm).

During *DS1* (ED2-ED4) NE cells originates the 1^st^ and 2^nd^ neuronal cohorts, i.e. afferent Mes5 neurons and efferent Stratum Griseum Centrale (SGC) neurons respectively. These neurons are born near the inner LM and then move radially towards the outer LM. By the end of DS1 (ED4) newly born SGC neurons form an incipient premigratory zone (PMZ) under the pial surface and begin their somal differentiation (Fig. 4, A-D). Simultaneously with the PMZ formation, the PNP gives rise to solid vascular sprouts that degrade the basal lamina (Fig. 5, A) and invade the outermost zone of the neuroepithelium (Fig. 5, B).

**Figure 6:**
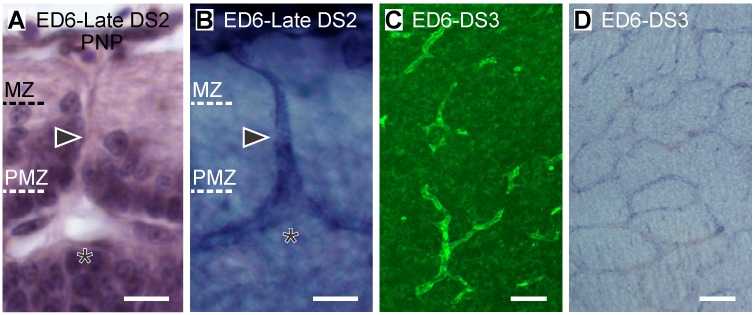
(A-B) Radial organization of the OT vasculature during late DS2 (ED6; HH29) (A: H-E; B: Diaphorase). Primitive radial vessels (arrowheads) bifurcate at the marginal zone (MZ)-premigratory zone (PMZ) interface or after entering the PMZ. Asterisks: bifurcation points. (C-D) Tangential organization of the PVP during DS3 (ED6; HH29) (Tangential sections; C: Notch D: Diaphorase). Radial vessels generate two to three terminal branches within the PMZ and form a tangentially oriented periventricular vascular plexus (Bars: A-B: 10 µm; C-D: 50 µm).

**Developmental Stage 2 (DS2)**. During *DS2* the PMZ thickens by the accumulation of postmitotic neurons at its innermost surface. These neurons correspond to the 3^rd^ neuronal cohort that will differentiate into the Stratum Griseum et Fibrosum Superficiale (SGFS) interneurons. At the same time, the 2^nd^ cohort of neurons, located at the outermost zone of the PMZ, originates a thick layer of bundles of axons that run tangentially under the pial surface and form the marginal zone (MZ). Simultaneously, the early vascular sprouts grow inwards, course across the MZ, invade the PMZ (Fig. 5, C-E) and form primitive short and straight radial vessels located at regular intervals. During this radial invasion, most vessels grow as cord-like structures formed by endothelial cells (Fig. 5, B and C). After entering the PMZ, the endothelial cells located at the tip of these cords form a lumen (Fig. 5, D and E) that gradually increases in diameter (Fig. 5, F-I). These primitive radial vessels do not branch while coursing through the MZ (Fig. 5, Dand E). However, during the late DS2, after arriving the MZ-PMZ interface or entering the PMZ, they form two or three oblique terminal branches. These branches appear as terminal bifurcations of radial vessels. Growing tips of these bifurcations are abundant within the PMZ or at the boundary between the PMZ and the generation zone (GZ). The GZ is composed of the ventricular zone (VZ) and the subventricular zone (sVZ) (Fig. 6, A and B).

**Figure 7:**
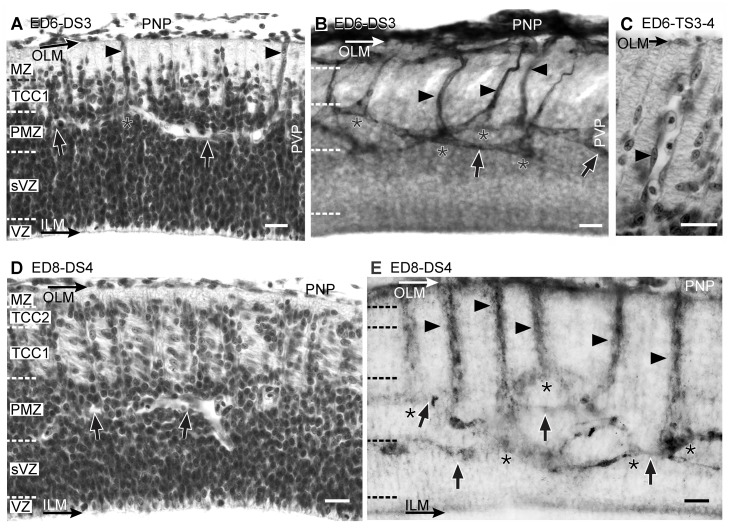
Radial organization of the OT vasculature during DS3 (A-B, ED6; HH29), TS3–4 (C, ED6, HH29) and DS4 (D-E: ED8; HH34). (A, C and D: H-E; B and E: Diaphorase). (A) The TCC1 appears between the marginal zone (MZ) and the premigratory zone (PMZ). (B) The primitive radial vessels (arrowheads) traverse the TCC1 and form the periventricular plexus (PVP). The tangential vessels of the PVP (arrows) display two preferential positions: most of them run through the TCC1-PMZ interface or through the PMZ-SVZ interface. Some of them run from one interface to the other. Asterisks: points of bifurcation. (C) During the TS-3–4, the primitive radial vessels (arrowhead) increase in length and diameter and are frequently occupied by several red blood cells (D) The TCC2 appears between the marginal zone (MZ) and the TCC1. Columns of radially migrating neurons can be seen at regular intervals traversing the TCC1. TCC1 neurons can be identified by their tangential orientation. (E) The OT cortex thickening is accompanied by the elongation of the radial vessels (arrowheads). Arrows: tangential vessels of the periventricular plexus; asterisks: bifurcations (Bars: 20 µm).

**Developmental Stage 3 (DS3)**. During *DS3*, the 2^nd^ cohort of neurons detaches from the PMZ and forms the TCC1 between the MZ and the PMZ. This DS is characterized by the disappearance of the vast majority of the growing tips of the terminal bifurcations. Instead, a periventricular vascular plexus (PVP) appears at the zone previously occupied by growing tips. A dynamic interpretation of the temporal sequence of images allows proposing that the PVP arises as a consequence of termino-terminal anastomoses between terminal bifurcations from neighboring primitive radial vessels. This early vascular organization, named as “primitive vascular plexus” is composed of the radial vessels and the PVP. Together they describe arch-shaped trajectories (Fig. 7, A and B). The PVP displays two preferential positions: some vessels run through the TCC1-PMZ interface while others run through the PMZ-sVZ interface. Some of them can be seen to run from one interface to the other (Fig. 7, B).

**Figure 8:**
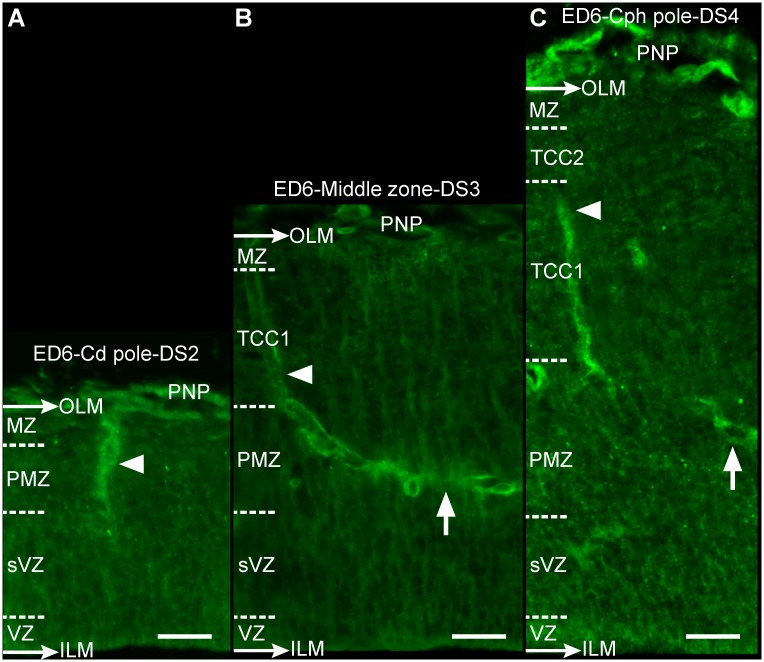
Space-dependent differences in the OT corticogenesis and vasculature development along the cph-cd axis (ED6; DS2-DS4; HH29) (A-C: Notch). (A) OT caudal pole: formation of primitive radial vessels (arrowheads); (B) Middle zone: generation of oblique or tangential terminal branches (arrows); and (C) OT cephalic pole: increase in complexity of the vascular network (Bars: 20 µm).

**Transitional Stage 3–4 (TS3–4)**. During *TS3–4* (ED6; HH29) the 3^rd^ neuronal cohort begins its radial migration towards the outer LM and the radial vessels significantly increase in length and diameter (Fig. 7,C). It is interesting that the following sequence of events: (*a*) vascular sprouting from the PNP, (*b*) primitive radial vessels formation and (*c*) PVP formation, begins at the cephalic regions and, from this point, progresses toward the caudal region. Between ED5 and ED6 (DS3/TS3–4), a cph-cd asymmetry becomes apparent in the spatial organization of the primitive vascular network. By ED6, the cph-cd asymmetry in the OT angiogenesis parallels the cph-cd asymmetry observed in the OT corticogenesis. Fig. 8, A-C shows the differences observed in the OT vasculature along the cph-cd axis; the differences in the OT cortex complexity are indicated on the left margin of each image. This asymmetry is also revealed by the spatial organization of the perineural plexus (See Spatial organization of the primitive radial vessels ingression into the OT cortex).

**Developmental Stage 4 (DS4)—Transitional Stage 4–5 (TS4–5)**. During *DS4* a critical mass of the 3^rd^ neuronal cohort surpasses the TCC1 and gives origin to the TCC2 between the MZ and the TCC1 (Fig. 7, D and E). During *TS4–5* (ED8–9), a subpopulation of the 3^rd^ neuronal cohort migrates radially, surpasses the TCC2, enter the deep region of the MZ and begin to form the TCC3. At the same time, the other TCCs thicken considerably due to the addition of migrating neurons proceeding from the sVZ. Simultaneously, obliquely oriented short lateral vessels sprout from the primitive radial vessels at the level of the TCC1 and TCC2.

**Figure 9:**
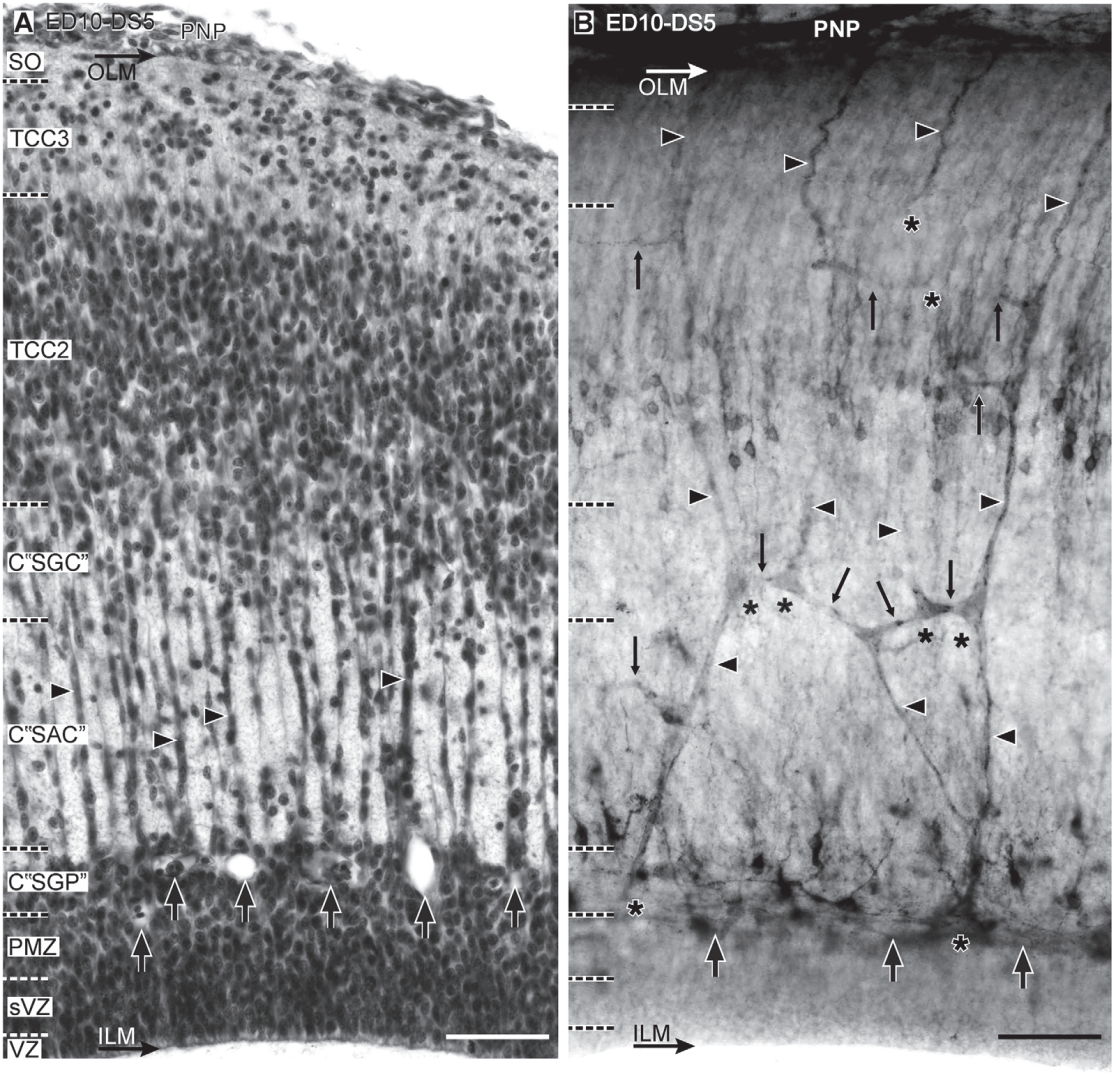
Radial organization of the OT vasculature during DS5 (ED10, HH36) (A: H-E; B: Diaphorase). (A) A well-defined TCC3 develops below the stratum opticum (SO). TCC1 delaminates into C “SGC”, C “SAC” and C “SGP”. (B) These changes are accompanied by the formation of short lateral branches and formation of different kind of anastomoses (thin arrows) between neighboring radial vessels. There is a decrease in the density of bifurcations at the periventricular plexus (thick arrows). Arrowhead: radial vessels; asterisk: bifurcations (Bars: 50 µm).

**Developmental Stage 5 (DS5)—Transitional Stage 5–6 (TS5–6)**. During *DS5* the TCC3 become a well-defined layer and the TCC1 delaminate into three new compartments: C “SGC”, C “SAC” and C “SGP” (Fig. 9, A). These changes in the cortex organization are accompanied by the production of lateral branches from the radial vessels at the level of almost every TCC (Fig. 9, B) and a slight decrease in the density of bifurcations at the periventricular plexus (See Influence of corticogenesis on the primitive vascular bed remodeling. Quantitative indexes). During *TS5–6* a subset of the 3^rd^ neuronal cohort migrates through the TCC2 towards a more superficial position and begins to form a poorly defined neuronal layer, the TCC4, below the TCC3. By this stage the NE cells proliferation declines, the radial migration across the C “SAC” continues and, consequently, the PMZ weakens. During this transitional period the OT vasculature does not change significantly.

**Figure 10:**
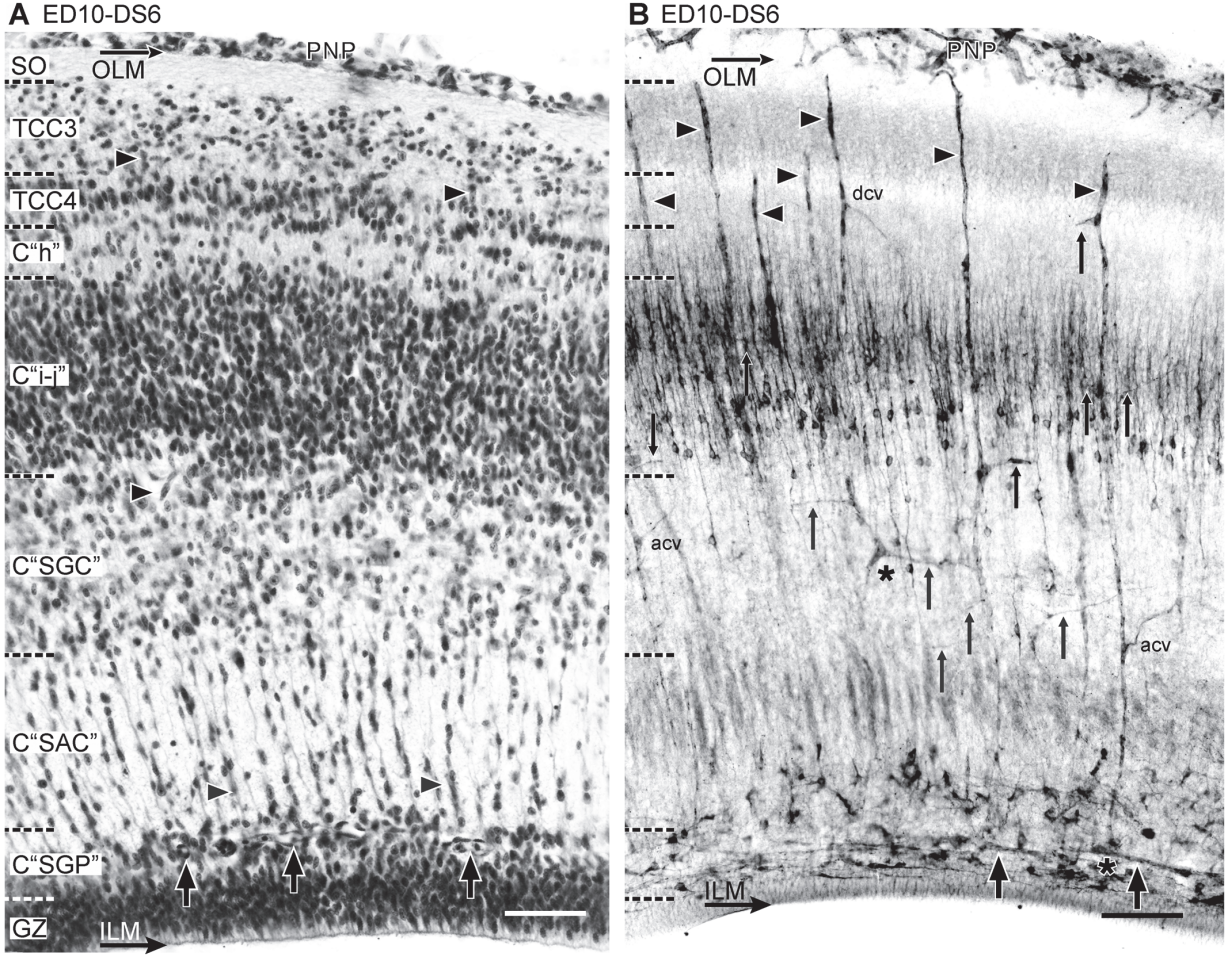
Radial organization of the OT vasculature during DS6 (ED10, HH36). (A: H-E; B: Diaphorase). (A) The TCC2 is no longer present because it delaminates into TCC4 -below TCC3-, C ‘‘h’’ and C ‘‘i-j”. The thickening of the OT cortex involves changes in the relative position of the different layers respect to each other and also respect to the original sites of lateral vessels origins. (B) The changes in the cortical organization are accompanied by sprouting of new branches at zones of high neuronal density and “pruning” of preexisting branches at zones of low neuronal density. Thin Arrows: lateral branches; arrowhead: radial vessel; dcv: descending branches; acv: ascending branches; thick arrows: periventricular plexus (Bars: 50 µm).

**Developmental Stage 6 (DS6)—Transitional Stage 6–7 (TS6–7)**. During *DS6* the TCC2 delaminates into three compartments: TCC4 and compartments C “h” and C “i-j” (Fig. 10, A-B). The C “h” can be identified as a band of low neuronal density, interposed between TCC4 and C “i-j”. C “h” later transforms into the definitive layer “h” of the SGFS. Another thin compartment of low neuronal density, the C “SFP”, can be distinguished between the future C “SFP” and the PMZ. This compartment can be identified topographically as the future fibrous layer named as SFP. Together, the C “SGP” and the C “SFP” will form the definitive SGFP. All these corticogenic changes are accompanied by a remodeling of the radial vessels branches: (a) sprouting of new branches at zones of new TCCs formation and (b) “pruning” of preexisting branches at zones where the density of neurons decline. During DS6, the NE cells proliferation declines significantly and this produces a decrease in the neuronal density at the PMZ and a significant reduction in the number of neurons migrating across C “SAC”. The density of lateral branches at the C “SGP” decreases while the density of bifurcations at the periventricular plexus remains unchanged with respect to DS5 (See Influence of corticogenesis on the primitive vascular bed remodeling. Quantitative indexes). During*TS6–7* the OT structure is better defined. The NE cells proliferation ceases, the GZ transforms into a pseudostratified-like epithelium and the PMZ almost completely disappears. Simultaneously, a sustained decrease in density of bifurcations and lateral branches occur at the periventricular plexus.

**Figure 11:**
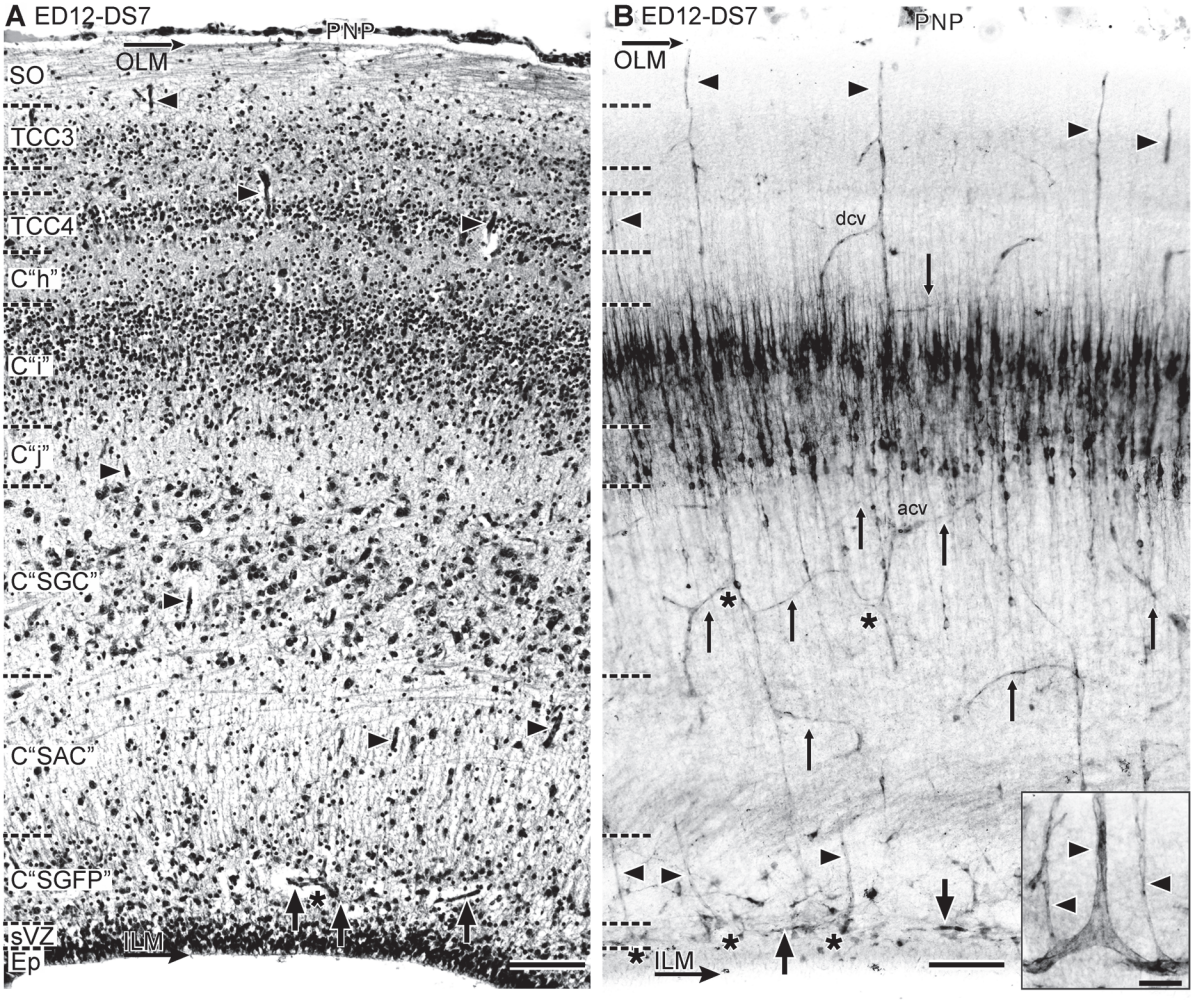
Radial organization of the OT vasculature during DS7 (ED12, HH38) (A: H-E; B: Diaphorase). (A) There is a remarkable thickening of the SO and the retinorecipient layers of the SGFS. (B) Significant changes in the vascular pattern accompany the retinorecipient layers remodeling and the late differentiation of the C “SGC”. Several different patterns of distribution of collateral branches and tangential to oblique anastomoses can be seen associated to the different TCCs. Inset: a new population of slender straight and radially ascending branches arises from the tangential vessels of the periventricular plexus (thick arrows). acv: ascending branches; dcv: descending branches; thin arrows: anastomoses; asterisk: bifurcations; arrowhead: radial vessel. The densely diaphorase-stained bands located halfway between the ventricular and the pial surface correspond to the layers of NOS+ neurons that typically populate the “i”—“j” complex at this stage [28] (Bars: A,B: 50 µm; Inset: 10 µm)

**Developmental Stage 7 (DS7)**. During *DS7* (ED12, HH38) the OT acquires its basic cortical organization. By ED12, the whole population of retinal ganglion cell axons has already entered the OT surface and the stratum opticum (SO) undergoes a remarkable thickening. Simultaneously, the TCC3, TCC4 and the fibrous layer-“Inter TCC3–4”- interposed between them undergo a remarkable thickening (Fig. 11, A-B). These three compartments will undergo a remodeling and will differentiate into the future retinorecipient layers of the SGFS. Significant modifications in the vascular pattern are associated to the remodeling of the retinorecipient layers (See Influence of corticogenesis on the primitive vascular bed remodeling. Quantitative indexes). Although the PMZ disappears, indicating that premigratory neurons are no longer present, the density of bifurcations at the periventricular plexus unexpectedly rises again. This phenomenon is probably associated to the onset of gliogenesis and glial cells differentiation. In fact, it is known that these events begin at ED12 [6, 9, 35]. It is interesting that during DS6 and DS7 a new population of branches appears: a specific kind of slender straight and radially ascending branches arises from the tangential vessels of the periventricular plexus (Fig. 11, B inset). The post-DS7 phases of the OT corticogenesis were described previously [3–5]. A summary of the major changes involved in corticogenesis (from DS1 to DS7) are presented in a single scheme in *Discussion*.

### Spatial organization of the primitive radial vessels ingression into the OT cortex

**Figure 12:**
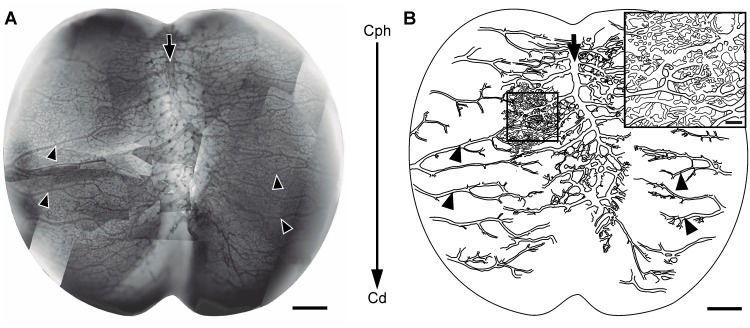
Tangential organization of the perineural plexus (ED7; HH31) (A: Diaphorase). (A) Image obtained by assembling several partial images of the OT leptomeningeal plexus. The superficial (venous) and the deepest (arterial) plexuses appeared overlapped. (B) Drawing of the largest vessels observed in “A”. The main venous vessels, running along the midline (arrows), and the main leptomeningeal arteries, running in a lateral-to-medial direction (arrowheads) exhibit a higher development in the cephalic region revealing the existence of a cph-cd angiogenic gradient (large arrow in the middle of both figures). The arrows and arrowheads shown in A and B allow the identification of the same vessels in both figures. Inset: Magnification of the box shown in figure “B” illustrating the complexity of the leptomeningeal capillary bed. (Bars: A,B: 500 µm; Inset: 100 µm).

**Figure 13:**
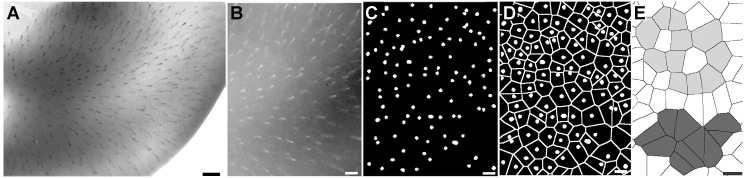
Spatial pattern of sites of radial vessels ingression into the OT cortex. (A) Image of the OT surface stained in toto with diaphorase. The site of ingression of each vessel can be clearly identified. (B) Negative image of the central zone of figure “A” shown at higher magnification. (C) Result of a digital image processing after binarization and segmentation of the image show in “B” in order to determine the sites of vessels ingression. (D) Demarcation of perivascular areas by means of the watershead transform. (E) Most vessels are typically surrounded by 6–8 vessels forming a honeycomb like organization (light gray). These “structured” areas are intermingled with “non-structured” areas where the 1–6 to 1–8 patterns are not observed (dark gray). (Bars: A: 200µm, B-E: 100µm).

Optic tecta stained *in toto* with diaphorase or with an anti-Notch antibody were used to analyze the spatial distribution of vessels over the OT tangential plane. Specimens stained *in toto* with the surrounding mesenchymal tissue, i.e. the future meningeal tissues, were used to analyze the spatial organization of the perineural plexus (Fig. 12). Specimens deprived of their overlaying perineural plexus were used to directly observe the sites of ingression of radial vessels over the OT pial surface. Images of the points of vessels ingression were recorded and analyzed by means of algorithms for digital image processing in order to define the borders and to estimate the extension of each perivascular area (Fig. 13).

Fig. 12,A shows a global image of the OT surface; it is a photographic composition obtained by assembling several partial images of the perineural plexus. The superficial venous and the deep arterial plexuses appear overlapped in the photograph. A cph-cd developmental gradient can be seen in the schematic representation of both plexuses drawn in fig. 12, B. The existence of a gradient is revealed by the distribution of values of inter-vessels intervals measured along the cph-cd axis. The length of inter-vessels intervals steadily increases along the cph-cd axis. The inset shown in fig. 12, B illustrates the complexity of the capillary bed interposed between the arterial and the venous vessels. The perineural vessels run from the cephalic-ventral-lateral region to the caudal-dorsal-medial one.

Fig. 13, A shows a photograph of the OT surface completely freed of the perineural plexus. The pattern of distribution of the superficial segments of the primitive radial vessels is easily appreciated in this kind of preparation. Fig. 13, B-D shows the result of a computer-assisted digital image processing of the image shown in fig. 13, A. This analysis clearly characterizes the variability in shape and size of the areas surrounding the radial vessels and their global pattern of distribution over the OT tangential plane. It can be seen that the global pattern includes different kinds of locally organized regions alternating with zones of random organization. In fact, the demarcation of perivascular areas (Fig. 13, E) reveals that most vessels are typically surrounded by 6–8 vessels forming a honeycomb like organization that cover an approximately polygonal area of around 0.01 mm^2^. These “structured” areas do not cover the entire tangential plane. They are intermingled with “non-structured” areas where the 1–6 to 1–8 patterns are not observed.

This analysis also allows estimating the variability of the zone of influence of each radial vessel, i.e. the area that surround each radial vessel estimated on the OT surface. Given that the diaphorase method labels the whole population of vessels (arteries + veins), analyses performed on OT stained *in toto* with diaphorase only allow approximate estimations of values (9640 µm^2^ ± 313 µm^2^). However, analyses performed on OT stained *in toto* with an anti-Notch antibody that specifically labels arterial vessels, allow estimating the variability of the periarterial area calculated on the OT surface (15161 µm^2^ ± 750 µm^2^).

### Influence of corticogenesis on the primitive vascular bed remodeling. Quantitative indexes.

**Figure 14:**
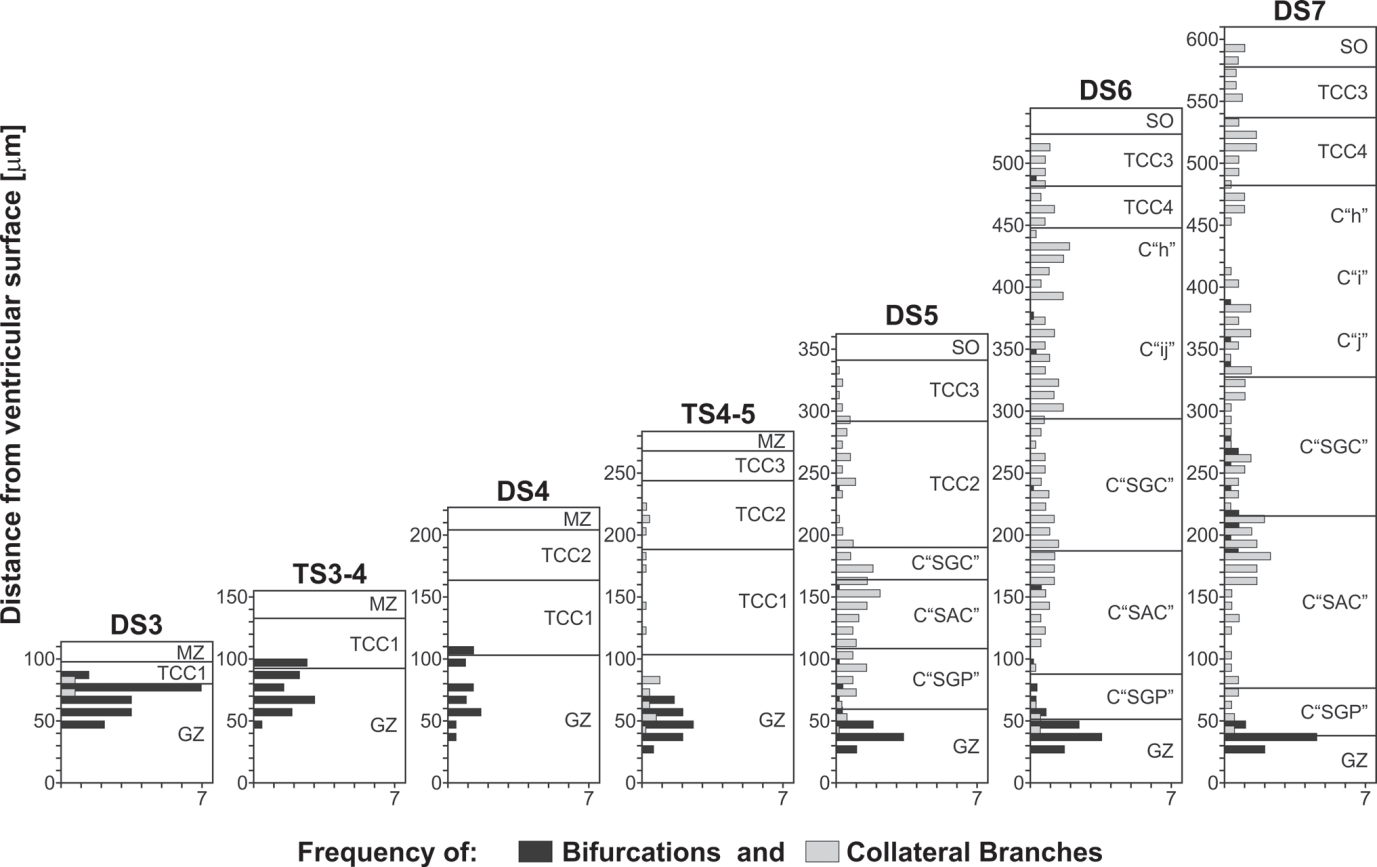
Changes in the local density of lateral branches and bifurcations as a function of both the position along the radial axis (y axis = distance from the ventricular surface) and the DSs. Each horizontal bar represents the density of lateral branches (gray bars) and bifurcations (black bars) calculated within 0.01 mm2 spatial windows distributed along the radial axis (Windows measure: 10 micrometer along the radial axis * 1 millimeter along the cephalic-caudal axis ). As NE cells proliferation decreases and postmitotic neurons leave the generation zone (GZ) and the premigratory zone (PMZ), the density of bifurcations (black bars) decreases. This change is associated with the disappearance of the PMZ and the decrease in cell proliferation at the GZ. During DS1-DS4 the OT wall irrigation depends on the primitive radial vessels. From DS4 onwards, different populations of new lateral branches appear at positions determined by the appearance of new TCCs.

**a) Changes in the local density of vessels branches and bifurcations along the radial axis.** Fig. 14 shows the variability in density of branches and bifurcations as a function of both the position along the radial axis and the DS. The bar graphs show that, *(a)* at any stage, the distribution of vessels along the radial axis is anisotropic, i.e. there are significant position-dependent differences in density of branches along the radial axis and *(b)* at any level of the radial axis, the density of branches changes as a function of the stage. This analysis shows a correlation between (a) the changes in density of branches and bifurcations along the radial axis and (b) the changes in the lamination patterns observed during successive stages.

**Figure 15:**
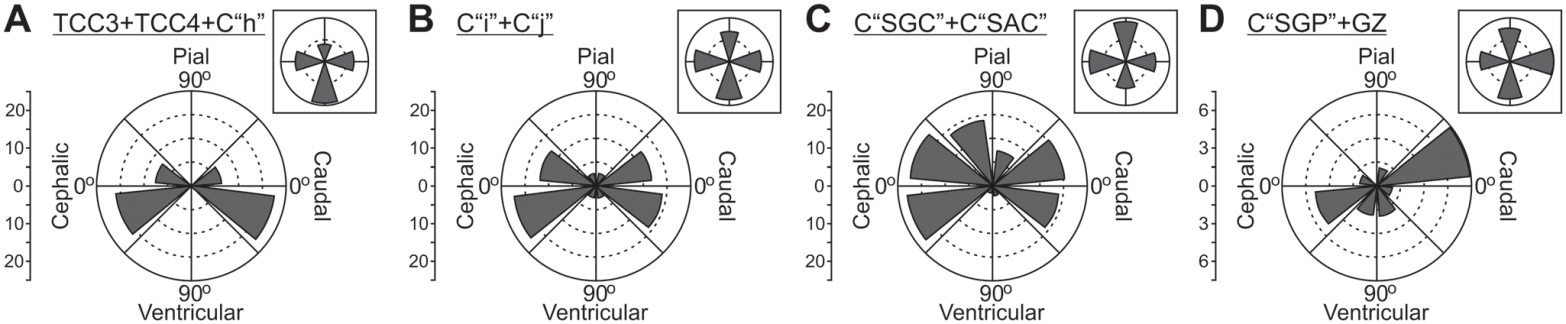
Direction of growth of vascular branches during (DS7; ED10; HH36). The polar coordinates systems show the preferential direction of growth of vascular branches growing from displayed by radial vessels’ lateral branches. (A-D) Records of lateral branches growth direction corresponding to different positions along the radial axis. The TCCs corresponding to each position are indicated on top of each graph. Inboxes summarize the preferential direction of growth in four quadrants. A clear anisotropy of growth direction is revealed.

**Figure 16:**
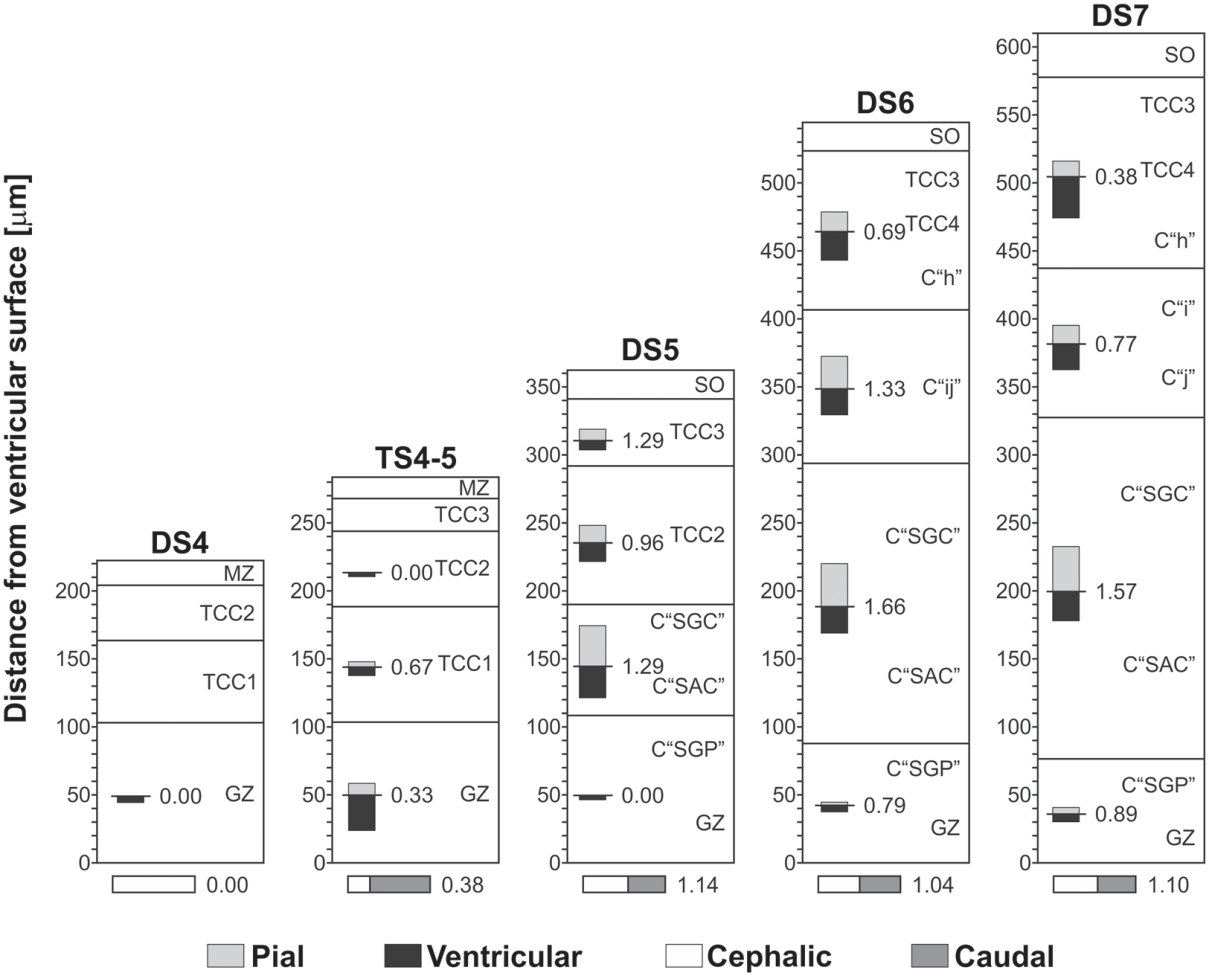
Analysis of anisotropy. The ratio “ascending vessels / descending vessels” is indicated for each TCC along the radial axis. Prior to DS5, vessels are mainly oriented towards the ventricular surface. By DS5, the ratio approximates 1. During DS6 and DS7, the ratio changes differentially as a function of the position along the radial axis or TCC. These changes show that the development of OT vasculature is strongly influenced by the OT corticogenesis since, as a rule the ratio increases in those zones where intensive proliferation, cell migration, dendritogenesis and synaptogenesis are taking place.

**b) Changes in the direction of growth of vascular branches and bifurcations along the radial axis.** Figs. 15 and 16 show the variability in the direction of growth of branches and bifurcations as a function of both the position along the radial axis and the DS. Polar co-ordinates graphs representing the directions of the branches arising from the radial vessels reveal anisotropies in this parameter along the radial axis. As an illustrative example, fig. 15, A-D shows polar co-ordinates graphs obtained at different levels (different TCCs) of the radial axis of an OT corresponding to DS7. The differences between the four graphs are clear indications of anisotropy. In order to represent such anisotropy in a simple way, the information contained in 16 polar co-ordinates graphs—corresponding to different DS and different positions along the radial axis- is summarized in Fig. 16. The anisotropy is expressed in terms of a quantitative index: the ratio between the number of vessels with ascending (pial) directions / number of vessels with descending (ventricular) direction. Note that, for an isotropic distribution the value of this ratio = 1. The graphs show that, with the exception of one case (the TCC2 during DS5, where the ratio = 0.96), the values of the ratio differ significantly from 1. The value of the ratio “number of branches with a cephalic direction / number of branches with caudal direction” is also indicated in fig. 16.

## DISCUSSION

The present paper shows that the OT angiogenesis is characterized by a sequence of typical developmental events that closely parallels the OT corticogenesis progression. These events can be divided into two phases. The first angiogenic phase leads to the formation of a primitive vascular bed and coincide with the early corticogenesis (DS1–DS3). These DSs corresponds to the generation and early migration of macroneurons precursors that transiently populate the PMZ and then form the TCC1. These neurons are precursors of the large efferent neurons of the SGC and SGP. During this phase the following sequence of angiogenic events takes place: (1) formation of the perineural plexus, (2) sprouting of vessels buds from the perineural plexus, (3) ingression and inward growth of primitive unbranched radial vessels, (4) generation of terminal branches in the deep layers (PMZ and VZ) and (5) formation of termino-terminal anastomoses between the terminal branches of the radial vessels. All these events lead to the formation of a primitive vascular network that expands between the pial surface and the VZ.

**Figure 17:**
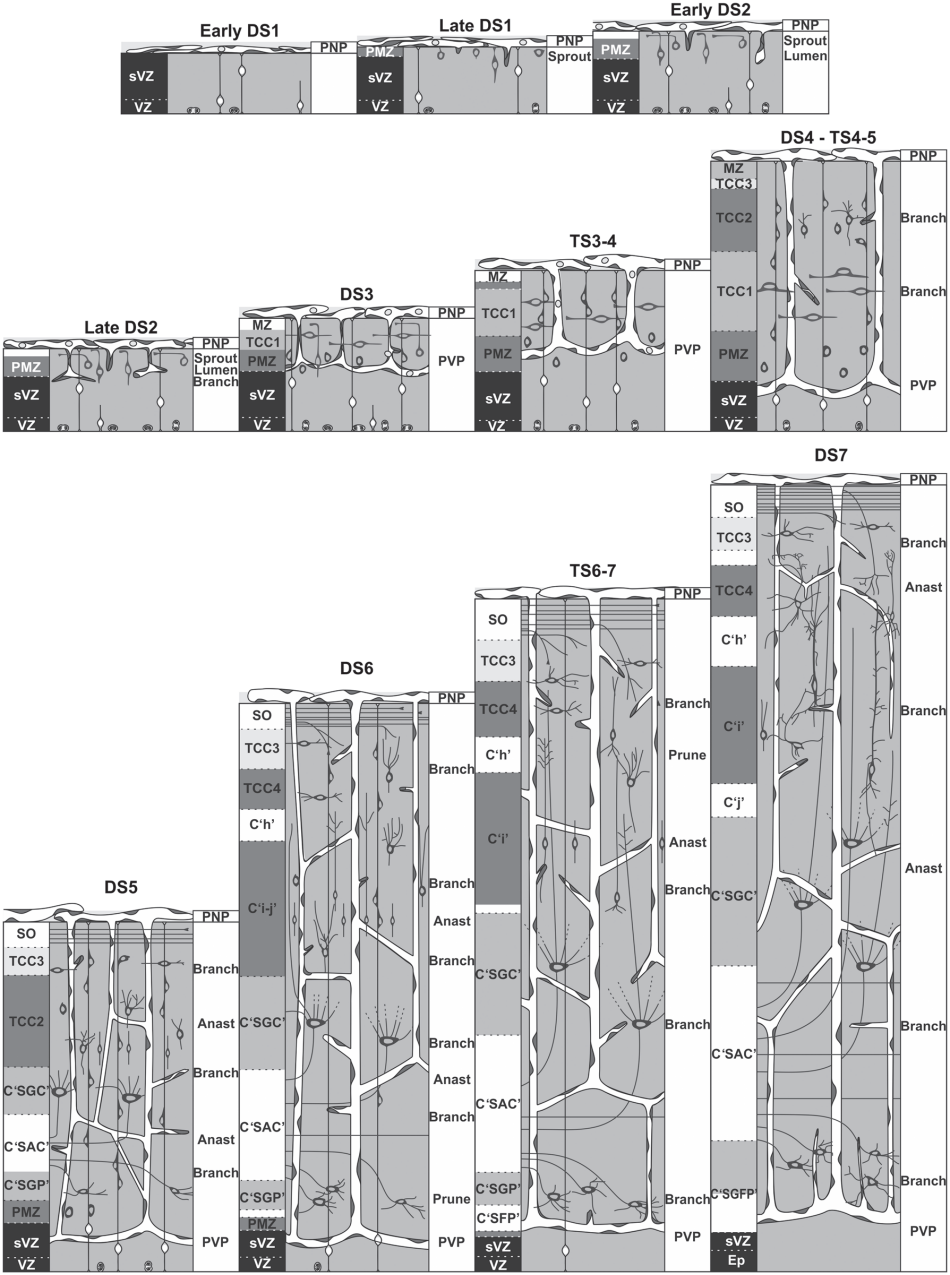
Schematic representation of the temporal-spatial correlation between OT angiogenesis and corticogenesis. OT angiogenesis is composed of two phases. The first phase (DS1-DS3) corresponds to the elaboration of a primitive vascular plexus associated to the generation of macroneurons and establishment of the deep OT layers (SGC and SGP). The second phase implies an intensive remodeling of the primitive vascular plexus and the establishment of the definitive OT vascular plexus. This phase is associated to the formation of the microneurons and its organization into the multilayered pattern of SGFS.

The second angiogenic phase involves a remodeling of the primitive vascular bed and the elaboration of a more complex and definitive vascular network. This phase coincides with the generation and postmitotic radial migration of the several subpopulations of microneurons that will form the SGFS (DS3 —DS7). This phase is characterized by a remarkable increase in complexity: elaboration of the multilayered organization of the SGFS, constitution of the stratum opticum and invasion of the most superficial (retinorecipient) layers by the retinal axons. All these events prepare the basic organization needed for the associative interneurons to elaborate the local circuit networks that functionally characterized the SGFS. The sequence of angiogenic events and their associations with the main corticogenic changes are summarized and schematically represented in fig. 17.

The present results show that, apart from the well-known influence of the dorsal-ventral and radial axes as reference systems for the spatial organization of OT angiogenesis, the cph-cd axis also exerts a significant asymmetric influence. This is evidenced by the fact that both the elaboration of the primitive vascular bed and its remodeling are temporally and spatially organized in parallel with the increase in complexity of the OT cortex. Similarly to OT corticogenesis, the entire process of OT angiogenesis progresses from a zone of maximal development (the site of retinal ganglion cell axons ingression) to a zone of minimal development (the caudal zone of the dorsal midline). Our results suggest the existence of control mechanisms that temporally synchronize and spatially adapt corticogenesis and angiogenesis. In this way, the increase in complexity observed in the vascular bed during development can be thought of as an adaptation of the developing vascular bed to an increasingly complex OT organization. This is not surprising since abundant evidence indicates that neuronogenesis and vasculo-angiogenesis are controlled and regulated by several cell signaling systems shared by developing neuroprogenitor cells and angiogenic precursor cells [36].

The term cortico-angiogenesis to describe the entire process is justified by the fact that tight correlations are found between specific corticogenic and angiogenic events, i.e., they take place simultaneously at the same position along the cph-cd and radial axes. As examples the following series of paired events can be cited:

(a) Production of vessels sprouting from the perineural plexus and invasion of the OT wall by newly formed primitive radial vessels begin simultaneously with the establishment of the PMZ. At that time the VZ displays the highest proliferating activity and the first cohort of migrating macroneurons start their differentiation at the PMZ (DS1, ED3, HH20).
(b) The terminal bifurcation of the primitive radial vessels, the establishment of termino-terminal anastomoses and formation of the periventricular plexus occurs specifically at the interfaces between TCC1-PMZ and between PMZ-GZ (DS3, ED6, HH29). These tangential planes correspond to zones of intense proliferation, perikarion differentiation and axonogenesis. All these activities are known to be high-energy consuming cell behaviors.
(c) The remodeling of the primitive vascular network occurs in parallel with changes in the cortex organization associated to cell proliferation, migration and differentiation: (1) By DS5, an increase in branches occurs at the C “SAC” (intensive migratory activity) and at the C “SGC” (rapidly differentiating neurons); 2) By DS6, an increase in lateral branches accompany the segregation of the TCC2 into TCC4, C “h” and C “i-j”, 3) Between DS6—DS7, the increase in neuronal density at the TCC4 and the decrease at the C “h” are accompanied by corresponding increase and decrease in lateral branches; beside, the C “SAC” (with actively migrating cells) and the C “SGC” (with intensive differentiation and neuritogenesis) show high density of branches. 4) By DS7, proliferation decreases at the GZ and its density of vascular bifurcations concomitantly declines.
(d) The anisotropic growth of lateral branches also correlates with histogenetic changes. In fact, the lateral branches of radial vessels that sprout during the remodeling of the PVP preferentially growth towards TCCs occupied by cells performing high-energy consuming activities (proliferation, migration, differentiation, neuritogenesis). An alternative explanation is that developing vessels adapt their direction of growth according to the relative displacement the developing TCCs underwent during corticogenesis. A future perspective of this work is to analyze the temporo-spatial pattern of expression of angiogenic and antiangiogenic factors in the developing OT and the role of developing neurons in the production of such factors.

## Supporting Information

S1 File Radial organization of the OT vasculature between DS3 and DS7. (A, C, E, G, I and K: H-E; B, D, F, H, J and L: Diaphorase). (A-B) ED6, DS3. The primitive radial vessels (arrowheads) traverse the TCC1 and form the periventricular plexus (PVP). Asterisks: points of bifurcation. (C-D). ED8, DS4. The OT cortex thickening is accompanied by the elongation of the radial vessels (arrowheads). Arrows: tangential vessels of the periventricular plexus; asterisks: bifurcations. (E-F). ED8, TS4–5. A population of short lateral vessels (asterisks) sprout laterally from the primitive radial vessels (arrowheads). (G-H) ED10, DS5. A well-defined TCC3 develops below the stratum opticum (SO). TCC1 delaminates into C “SGC”, C “SAC” and C “SGP”. These changes are accompanied by the formation of short lateral branches and formation of different kind of anastomoses (thin arrows) between neighboring radial vessels. There is a decrease in the density of bifurcations at the periventricular plexus (thick arrows). Arrowhead: radial vessels; asterisk: bifurcations. (I-J) ED10, DS6. The changes in the cortical organization are accompanied by sprouting of new branches at zones of high neuronal density and “pruning” of preexisting branches at zones of low neuronal density. Thin Arrows: lateral branches; arrowhead: radial vessel; dcv: descending branches; acv: ascending branches; thick arrows: periventricular plexus. (K-L) ED12, DS7. Significant changes in the vascular pattern accompany the retinorecipient layers remodeling and the late differentiation of the C “SGC”. Several different patterns of distribution of collateral branches and tangential to oblique anastomoses can be seen associated to the different TCCs. Inset: a new population of slender straight and radially ascending branches arises from the tangential vessels of the periventricular plexus (thick arrows). acv: ascending branches; dcv: descending branches; thin arrows: anastomoses; asterisk: bifurcations; arrowhead: radial vessel. (Bars: 20 µm).
(TIF)

S2 File Original unmodified images files of diaphorase preparations for DS3 to DS7. (TIF)
