# Correction: Temporal-Spatial Correlation between Angiogenesis and Corticogenesis in the Developing Chick Optic Tectum

**DOI:** 10.1371/journal.pone.0122549

**Published:** 2015-03-19

**Authors:** The PLOS ONE Staff

The first and fifth author’s names are spelled incorrectly. The first author’s correct name is: Alejandra Rodriguez Celin. The fifth author’s correct name is: Sara Fiszer de Plazas.

The correct citation is: Rodriguez Celin A, Rapacioli M, Gonzalez MA, Ballarin VL, Fiszer de Plazas S, López-Costa JJ, et al. (2015) Temporal-Spatial Correlation between Angiogenesis and Corticogenesis in the Developing Chick Optic Tectum. PLoS ONE 10(1): e0116343. doi:10.1371/journal.pone.0116343
