# Supplementary figures and images for: Temporal-Spatial Correlation between Angiogenesis and Corticogenesis in the Developing Chick Optic Tectum
Source: PLoS One. 2015 Jan 29;10(1):e0116343. doi: 10.1371/journal.pone.0116343 (PMC4310613; doi:10.1371/journal.pone.0116343)

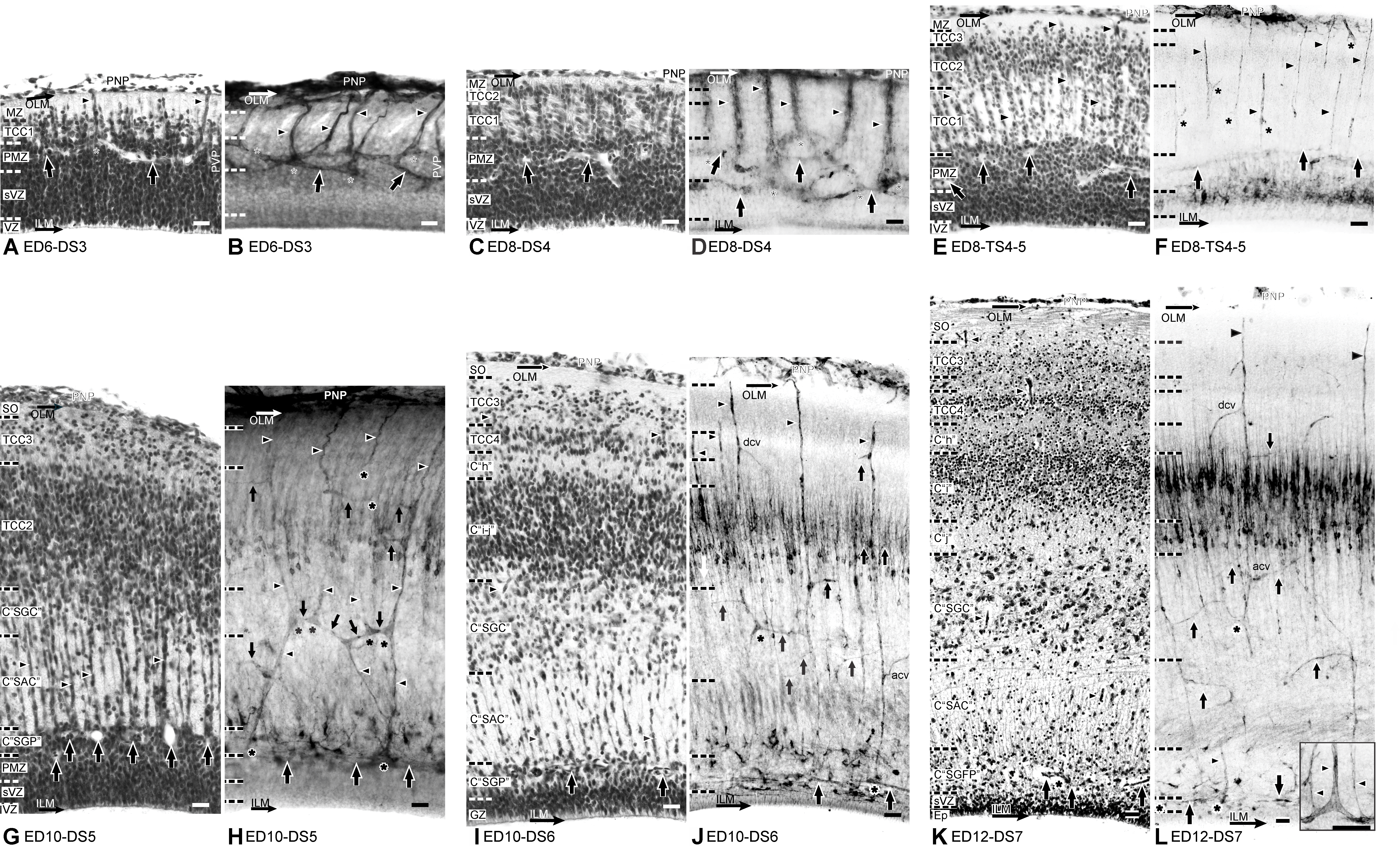

Supplement: S1 File — (A, C, E, G, I and K: H-E; B, D, F, H, J and L: Diaphorase). (A-B) ED6, DS3. The primitive radial vessels (arrowheads) traverse the TCC1 and form the periventricular plexus (PVP). Asterisks: points of bifurcation. (C-D). ED8, DS4. The OT cortex thickening is accompanied by the elongation of the radial vessels (arrowheads). Arrows: tangential vessels of the periventricular plexus; asterisks: bifurcations. (E-F). ED8, TS4–5. A population of short lateral vessels (asterisks) sprout laterally from the primitive radial vessels (arrowheads). (G-H) ED10, DS5. A well-defined TCC3 develops below the stratum opticum (SO). TCC1 delaminates into C “SGC”, C “SAC” and C “SGP”. These changes are accompanied by the formation of short lateral branches and formation of different kind of anastomoses (thin arrows) between neighboring radial vessels. There is a decrease in the density of bifurcations at the periventricular plexus (thick arrows). Arrowhead: radial vessels; asterisk: bifurcations. (I-J) ED10, DS6. The changes in the cortical organization are accompanied by sprouting of new branches at zones of high neuronal density and “pruning” of preexisting branches at zones of low neuronal density. Thin Arrows: lateral branches; arrowhead: radial vessel; dcv: descending branches; acv: ascending branches; thick arrows: periventricular plexus. (K-L) ED12, DS7. Significant changes in the vascular pattern accompany the retinorecipient layers remodeling and the late differentiation of the C “SGC”. Several different patterns of distribution of collateral branches and tangential to oblique anastomoses can be seen associated to the different TCCs. Inset: a new population of slender straight and radially ascending branches arises from the tangential vessels of the periventricular plexus (thick arrows). acv: ascending branches; dcv: descending branches; thin arrows: anastomoses; asterisk: bifurcations; arrowhead: radial vessel. (Bars: 20 µm). (TIF) [file pone.0116343.s001.tif]

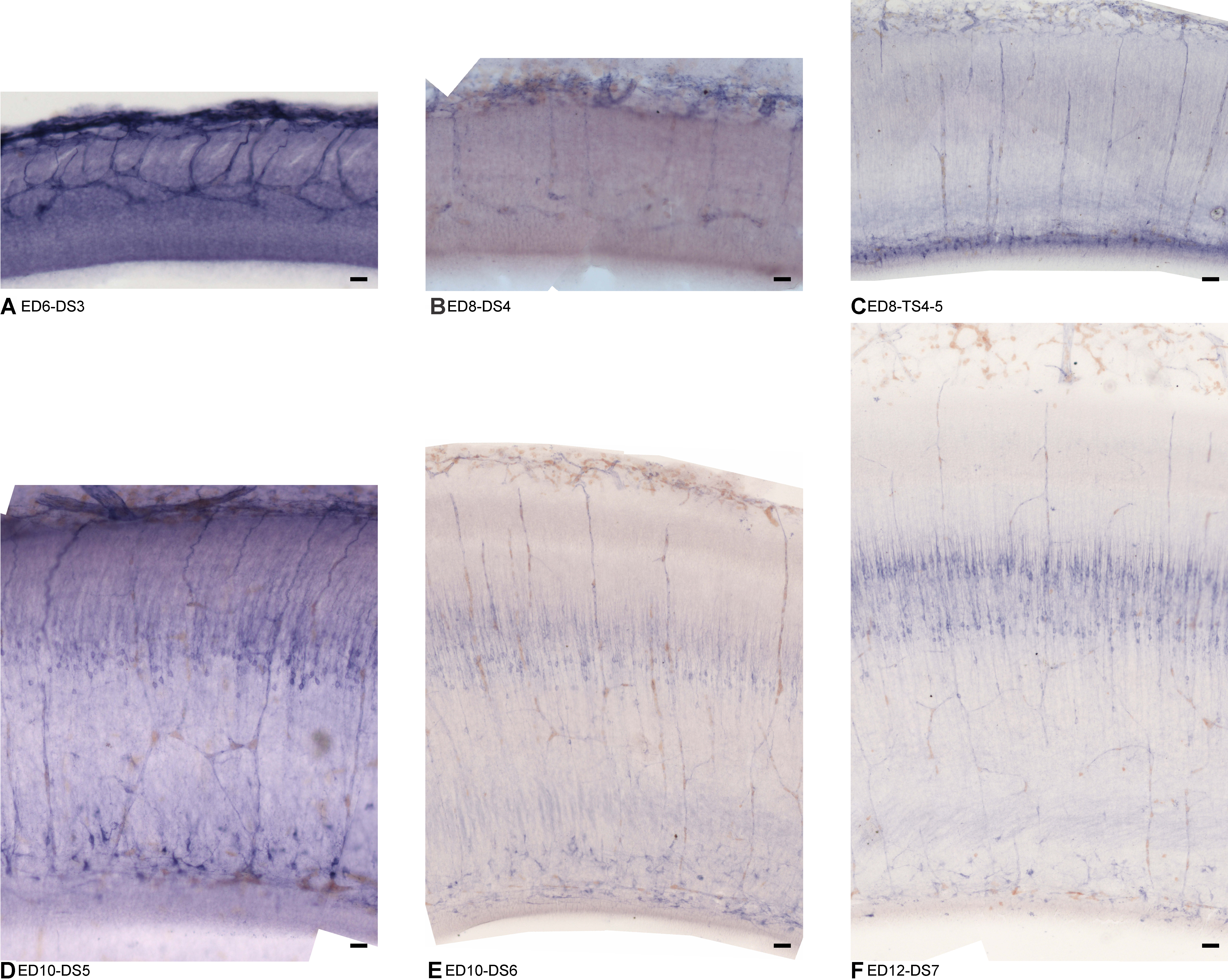

Supplement: S2 File — (TIF) [file pone.0116343.s002.tif]
